# Rationale and Design of a Study to Assess the Engagement and Usefulness of the Care4Today Connect Digital Health Application for Disease Management in Coronary Artery Disease and Peripheral Artery Disease (iPACE‐CVD Study)

**DOI:** 10.1002/clc.70039

**Published:** 2024-12-11

**Authors:** Dhanunjaya Lakkireddy, Dominick J. Angiolillo, Kristofer Charlton‐Ouw, Brian Jefferson, Syed Peeran, Mohannad Bisharat, Luis Ortega‐Paz, Ante Harxhi, Simrati Kaul, Evelyne Michaud, Stephanie Juan, Breeana Woods, CV Damaraju, Gregory Fontana, Marc P. Bonaca

**Affiliations:** ^1^ Kansas City Heart Rhythm Institute HCA Midwest Health Overland Park Kansas USA; ^2^ Division of Cardiology University of Florida College of Medicine Jacksonville Florida USA; ^3^ Department of Clinical Sciences University of Houston College of Medicine Houston Texas USA; ^4^ Tristar Centennial Medical Center Nashville Tennessee USA; ^5^ Coastal Cardiothoracic and Vascular Surgery Portsmouth Regional Hospital Portsmouth New Hampshire USA; ^6^ Ashchi Heart and Vascular Center and HCA Florida Memorial Hospital Jacksonville Florida USA; ^7^ Janssen Scientific Affairs, LLC, a Johnson & Johnson Company Titusville New Jersey USA; ^8^ Johnson & Johnson Technology Solutions Raritan New Jersey USA; ^9^ Cardiovascular Institute of Los Robles Health System HCA Healthcare Research Institute Thousand Oaks California USA; ^10^ CPC Clinical Research, Department of Medicine University of Colorado Aurora Colorado USA

**Keywords:** cardiovascular disease, health tracker, medication adherence, mobile health (mHealth), patient education, patient engagement, smartphone application

## Abstract

**Introduction:**

Coronary artery disease (CAD) and peripheral artery disease (PAD) increase the risks of cardiovascular events and death. Digital health technologies are rapidly expanding to improve healthcare quality and access. The Care4Today Connect (C4T CAD‐PAD) mobile application is designed to help patients with CAD and/or PAD improve medication adherence, learn about their disease, make lifestyle modifications, and enhance healthcare provider (HCP) connection via an HCP‐facing portal.

**Hypothesis & Methods:**

The prospective, single‐arm, multicenter, noninterventional iPACE‐CVD (innovative Patient compAnion impaCting health outcomEs: a CardioVascular Digital health program) study (ClinicalTrials.gov identifier: NCT06052319) is evaluating engagement and usefulness of the application for patients with CAD and/or PAD in clinical settings. Application access is provided with a code from patients' HCPs. Key features include medication and health experience tracking. The application is available in English and Spanish and for iOS and Android devices. Engagement is defined as the proportion of patients who use the application for ≥ 10 weeks during the 3‐month study period. Application use is defined as the number of patients using ≥ 1 application feature(s) each week. Usefulness is determined by the percentage of engaged patients who complete the My Feedback Matters survey with a satisfaction response score of > 2 (on a 5‐point scale, where 1 = *strongly disagree* and 5 = *strongly agree*) for at least three of the six questions.

**Results:**

A total of 271 participants were enrolled between November 29, 2023, and May 15, 2024. The study concluded on August 15, 2024.

**Conclusion:**

This study will help enhance the application for subsequent studies. **Trial Registration:** NCT06052319

## Summary

- The Care4Today Connect (C4T CAD‐PAD) platform features a digital smartphone application designed to help patients with coronary artery disease (CAD) and/or peripheral artery disease (PAD) achieve the following: (1) improve medication adherence; (2) acquire knowledge about their disease; (3) embark on lifestyle modifications; (4) track their health; and (5) increase their connection to their healthcare providers.
- The C4T CAD‐PAD application is undergoing an optimization study (iPACE‐CVD) to determine engagement and usefulness in patients with CAD and/or PAD in clinical settings.
- Digital health initiatives that can continuously engage patients have the potential to improve health outcomes and enhance partnerships between patients and their healthcare providers.

## Graphical abstract

The Care4Today Connect CAD‐PAD (C4T CAD‐PAD) platform features a patient‐facing digital smartphone application that is designed to help patients with disease management. The application includes features to help with the following: (1) improve medication adherence; (2) acquire knowledge about their disease; (3) embark on lifestyle modifications; (4) track their health; and (5) increase their connection to their healthcare providers. The iPACE‐CVD (innovative Patient compAnion impaCting health outcomEs: a CardioVascular Digital health program) study (ClinicalTrials.gov identifier, NCT06052319) will assess the engagement and usefulness of the C4T CAD‐PAD smartphone application in patients with coronary artery disease (CAD) and/or peripheral artery disease (PAD).

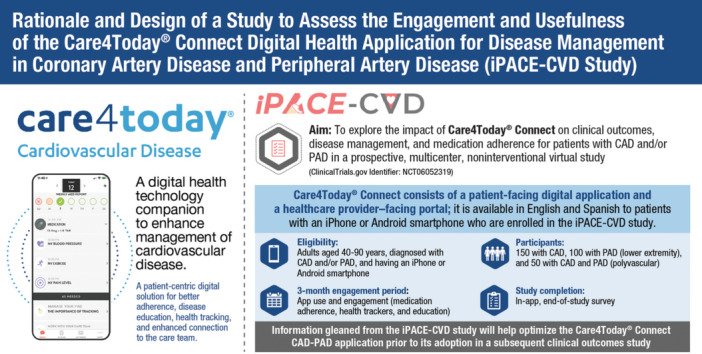

**Abbreviations**
App: application
C4T: Care4Today
CAD: coronary artery disease
HCP: healthcare provider
ICF: informed consent form
PAD: peripheral artery disease

## 1 Introduction

Coronary artery disease (CAD) and peripheral artery disease (PAD) share risk factors, including lifestyle behaviors (smoking, physical activity, diet, and weight) and health factors (cholesterol levels and blood pressure) [1, 2]. Up to 42% of patients with CAD also have PAD, and the presence of both CAD and PAD further increases the risk of major adverse cardiac events and lower extremity revascularization [3, 4]. Treatment goals for CAD and PAD are aimed at reducing symptoms and preventing future cardiovascular events [5, 6]. Cardiovascular disease burden is even greater in some racial and ethnic minority groups that have not only a higher disease prevalence but also more severe disease and increased risk of adverse events [2, 7, 8, 9, 10, 11, 12]. Addressing cardiovascular risks and disparities among racial and ethnic groups remains an unmet need [13, 14].

Digital health technologies may empower patients to become active participants in their own care through programs that are designed to improve the quality, access, and efficacy of healthcare information [15, 16]. However, there is limited information on digital technologies for cardiovascular disease, particularly for patients with CAD and PAD [17, 18]. To this extent, the Care4Today Connect CAD‐PAD (C4T CAD‐PAD) platform features a patient‐facing digital smartphone application that is designed to help patients with the following: (1) improve medication adherence; (2) acquire knowledge about their disease; (3) embark on lifestyle modifications; (4) track their health; and (5) increase their connection to their healthcare providers (HCPs). The solution allows for the flow of patient‐reported information to their healthcare team via an HCP‐facing portal. Given that this is the first of its kind, there is no information on the usefulness and performance of this type of application regarding patient engagement and satisfaction or the impact of the application on outcomes and quality of life.

The iPACE‐CVD (innovative Patient compAnion impaCting health outcomEs: a CardioVascular Digital health program) study (ClinicalTrials.gov identifier, NCT06052319) will assess engagement and usefulness of the C4T CAD‐PAD smartphone application in patients with CAD and/or PAD.

## 2 Materials and Methods

### 2.1 Study Design and Digital Health Application

**Figure 1:**
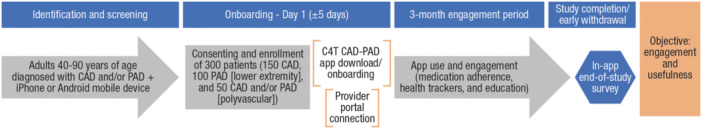
Study design of the C4T CAD‐PAD optimization study. App, application; C4T, Care4Today; CAD, coronary artery disease; PAD, peripheral artery disease.

This is a prospective, single‐arm, multicenter (six sites), noninterventional study to assess the C4T CAD‐PAD smartphone application in clinical practice (Figure 1). The study will enroll approximately 150 patients with CAD, 100 with PAD, and 50 with CAD and PAD (polyvascular) based on their most recent diagnoses. Patients will choose to use the C4T CAD‐PAD application over a 3‐month period. All patients must sign an informed consent form (ICF) allowing data reporting and collection in accordance with local requirements and/or the study protocol. The ICF will be completed in the physician's office or remotely (via phone and online portal) and informs patients that no medical care will be provided by the application and that they should consult with their physician for medical advice. The study will be completed virtually, and there is no requirement for the patients to visit a study site. During the virtual onboarding process, patients will be asked to agree to the application's terms of use, privacy policy, and the statement of health information disclosure.

Patients will join the study on their smartphone by using an access code provided by the study site. This code places patients into the C4T CAD‐PAD care module, which is further customized by disease (CAD, PAD, or both), where patients can set a health goal. The site code uniquely connects the patient to their study site via the HCP care portal. Educational articles available to patients will vary based on their designated content module (CAD, PAD, or both). All patients in the study will accumulate and receive points as reimbursement for their time completing study tasks while on the study, the balance of which they will be able to view in the app. The number of points earned by patients throughout the 3‐month engagement period will vary based on their level of involvement in completing applicable engagement activities whether assigned to them or self‐selected by the patient. The points earned during the study will be redeemable at the end of the study.

**Figure 2:**
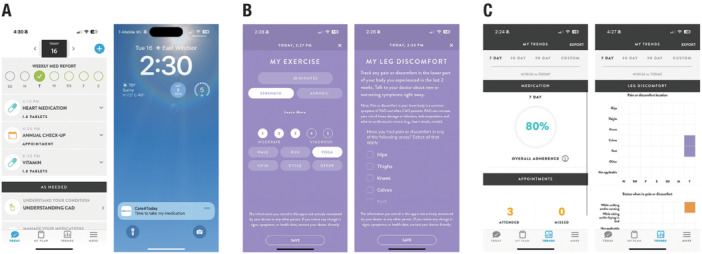
C4T CAD‐PAD screen captures of (A) medication and appointment reminders, (B) health and lifestyle behavior trackers, and (C) easy‐to‐follow trend reports. C4T, Care4Today; CAD, coronary artery disease; PAD, peripheral artery disease.

Patients will use the C4T CAD‐PAD application on a daily or as‐needed basis during the study period to record information on medications, healthcare appointments, health metrics, and lifestyle behaviors as shown in Table 1. The digital health application includes trackers and easy‐to‐follow trend reports for medication adherence, healthcare appointment attendance, and health and lifestyle behaviors (Figure 2).

**Table 1:** Information collected in the C4T CAD‐PAD digital health application used to measure engagement and usefulness.

Information category	Recorded measures
Medication adherence tracker	Medication reminders Missed medications Medication refills
Appointment tracker	Healthcare appointment attendance
Health metrics	Blood pressure Heart rate Blood sugar Hemoglobin A1c Lipid levels
Lifestyle behaviors	Daily routine/function Energy level Smoking (cigarette use tracker) Activity level Sleep Mood Meal rater Leg cramp/discomfort^a^ Encouragement Exercise Daily check‐in Step count Weight Water intake Sodium intake Pain level Nausea

Abbreviations: C4T, Care4Today; CAD, coronary artery disease; PAD, peripheral artery disease.

^a^ The leg cramp/discomfort measure is not limited to patients with PAD and can be recorded by all patients in the study as CAD and PAD often occur together.

The C4T CAD‐PAD application includes both scheduled and daily application‐based notification reminders. It is available for both iPhone (iOS) and Android smartphones and in English and Spanish languages. On or after Day 75, patients will be asked to complete a survey to assess overall satisfaction with the application features and educational content. The overall duration of the study for an individual patient is expected to be approximately 3 months.

### 2.2 Patient Eligibility

Participating sites will be encouraged to identify eligible patients with CAD and/or PAD based on the inclusion and exclusion criteria listed in Table 2. Male or female patients who are 40−90 years of age (inclusive), have a diagnosis of CAD and/or PAD, and are receiving therapy or about to initiate therapy are eligible. They must own a smartphone, either an iPhone (iPhone 6S or newer; iPhone SE [any generation]) or Android (Android 23, released 2015 or later) and have an active email account. They must sign a participation agreement/ICF allowing data collection in accordance with local requirements and be willing to download and use the C4T CAD‐PAD application. Patients with any cardiovascular or noncardiovascular condition with a poor prognosis, as assessed by the investigator, are not eligible to participate in the study.

**Table 2:** Inclusion and exclusion criteria for participants in the study.

Inclusion criteria	Exclusion criteria
Male or female aged 40−90 years, inclusive Must have a diagnosis of CAD and/or PAD and are on therapy or about to initiate therapy Own a smartphone: ○ iPhone 6s or newer ○ iPhone SE (any generation) ○ Android 23, released in 2015 or later Have an active email account Must sign a participation agreement/ICF allowing data collection in accordance with local requirements Willing to download and use the C4T CAD‐PAD application	Any cardiovascular conditions (e.g., recent stroke, high bleeding risk, and severe heart failure) or noncardiovascular condition deemed as poor prognosis by the investigator and which may prevent a patient from completing the study Unable to read/write English or Spanish Visual/hearing impairment or mental disability that would preclude independent application use Patients currently using the C4T Connect application before signing the ICF

Abbreviations: C4T, Care4Today; CAD, coronary artery disease; ICF, informed consent form; PAD, peripheral artery disease.

### 2.3 Outcomes

The primary objectives of this study are: (1) to assess the C4T CAD‐PAD application patient engagement and (2) to understand the usefulness of the C4T CAD‐PAD application to the patients. Patient engagement will be assessed as the proportion of participating patients who use the application for ≥ 10 weeks (~80% of the time) during the 3‐month study period. Weekly application use is defined as the number of patients using ≥ 1 feature of the application once a week. A distribution of participants using more than 1 feature of the application at least once a week will be presented and explored against the current definition of weekly application use. Usefulness will be determined by the percentage of engaged patients who complete the end‐of‐study survey with a satisfaction response score of > 2 (on a 5‐point scale, defined as: 1 = *strongly disagree*, 2 = *disagree*, 3 = *neutral*, 4 = *agree*, and 5 = *strongly agree*) for at least three of the six questions.

Data will be collected directly from the application for all patients by the application developer (Johnson & Johnson Services Inc.). Source data from the application will be made available to the participating clinical sites via the provider portal. All personal information will be deidentified/anonymized before upload to a secure portal for study analysis. The key parameters will include medication adherence, health metrics, and lifestyle behaviors (Table 1). No safety data are collected by study design and, therefore, no safety data analysis will be performed. However, during the study, if a site becomes aware of any incidental safety data involving a Janssen medicinal product, the data will be collected and spontaneously reported.

### 2.4 Statistical Methods

The objective of this study is to explore the engagement and usefulness of the C4T CAD‐PAD digital health application among patients with CAD and/or PAD. In line with pilot investigations, no formal hypothesis has been specified and no formal sample size calculation was conducted [19, 20]. Enrollment of approximately 150 patients with CAD, 100 with PAD, and 50 with both CAD and PAD (polyvascular) is planned to sample a substantial number of representative patients from the broad spectrum of the CAD and/or PAD population and is considered reasonable to meet the exploratory objectives. Because many patients with PAD also have CAD, the PAD and polyvascular groups may be analyzed together in the event that there are challenges in enrolling sufficient numbers of patients with PAD alone.

Analyses of application data will include all patients enrolled in the study and given access to the digital health application. The observation period for data analysis will be from the time of enrollment into the study until the end of the study (3 months or early withdrawal). The main analysis will be using data from all patients obtained through the full observation period of 3 months. Qualitative variables will be summarized by their frequency distributions (counts and percentages) and quantitative variables by their means and standard deviations. Continuous nonnormally distributed variables will be summarized by medians and interquartile ranges and tested for any significant nonnormality using the Kolmogorov−Smirnov test. Where appropriate, such nonnormal data will also be considered for transformation (e.g., logarithmic) before the analysis. The overall percentage of application users in the study by disease diagnosis type will be provided. Point estimates will be provided, as necessary, along with their 95% confidence intervals.

### 2.5 Study Organization

This study is sponsored by Janssen Scientific Affairs, LLC. An independent steering committee composed of members including sponsor representatives, experts in the management of CAD‐PAD patients, experts in digital health technology, and advocacy representatives provided oversight.

## 3 Results

Patients were enrolled from November 29, 2023, to May 15, 2024. A total of 271 patients were enrolled. Of these, 59% were male, 40% were female, and 1% preferred not to disclose their sex. The study was completed on August 15, 2024.

## 4 Discussion

This report describes the rationale and design of the C4T CAD‐PAD application optimization study that will assess the engagement and usefulness of the digital health application in patients with CAD and/or PAD. This application is innovative for cardiovascular disease, with the goal of tracking medication adherence, health metrics, and lifestyle behaviors in a single application that is dedicated to supporting patients with CAD and/or PAD. C4T CAD‐PAD is available for both major smartphone operating systems (iOS and Android) and in English and Spanish, allowing for widespread use across patient demographic groups in the United States, including underserved communities. The results of this optimization study will inform researchers about application engagement and usefulness based on patients' utilization and assessment of the application.

Digital health initiatives and interventions are gaining momentum in the cardiovascular disease space [21]. A review of digital health interventions for patients with atherosclerotic‐related cardiovascular diseases was conducted in 2020 and evaluated health disparity populations [17]. A total of 38 studies were identified, of which 16 evaluated telemedicine and 14 evaluated mobile health technologies that leveraged mobile telephones to send/receive text messages, utilized global positioning systems, or were smartphone applications. The mobile health technologies were designed to support medication adherence and blood pressure monitoring and provide health information. Statistically significant improvements in health outcomes were observed in most mobile health technology studies (12/14), primarily among patients with hypertension, diabetes and hypertension, or heart failure [17]. For these patients with chronic conditions, digital health technologies available through smart devices may support long‐term behavioral changes, including medication adherence, and attenuate health disparities given the widespread use of smartphones, especially when office visits are limited. Furthermore, medication adherence is highly associated with clinical and economic outcomes that impact long‐term mortality risk, especially in patients with chronic diseases [22, 23, 24]. Importantly, a study of a digital health intervention for use after acute myocardial infarction indicated that these tools may contribute to achieving healthcare equity because it found similar use by age, sex, and race [25].

The clinical implications of digital health applications, such as C4T CAD‐PAD, have the potential to be widespread by offering patients a means to identify and manage their cardiovascular disease risks and engage in their health management. The digital health application provides patients with a platform to report their health trends to their HCP between office visits for review in real‐time or for discussion at their next visit. In addition, the C4T CAD‐PAD application serves as a digital pillbox for patients, with the ability to track their own medications and schedule reminders for daily dosing and prescription refills. The ability to engage patients in their own healthcare is crucial to achieving improved health outcomes and quality of life while reducing healthcare resource utilization and costs [16]. The use of digital health applications may lead to a better partnership between patients, their families, and HCPs, encouraging healthy behaviors by tracking medications and health status. The results of this optimization study of the C4T CAD‐PAD application will provide important information to enhance the application based on patients' feedback and application utilization in a subsequent study to assess clinical outcomes.

### 4.1 Study Limitations

The C4T CAD‐PAD application is not available as a web‐based desktop software for patients, and there is only a web‐based care portal available for HCPs. With smartphone ownership in the United States ranging from 97% in people aged 30−49 years to 76% in people aged ≥ 65 years, it is likely that many eligible patients will be comfortable engaging with a digital application [26]. It is possible that there will be a subset of patients who will be less familiar with technology or face barriers to access, including smartphone ownership and email access, which may prevent them from enrolling in the study. However, careful design of the study and the technology may mitigate these issues [27].

This is an observational study without a control arm; therefore, only descriptive information on the enrolled population will be provided. Short‐term follow‐up (3 months) may make it difficult to observe changes in medication adherence and lifestyle behaviors. Because this is a virtual study, entries may be completed by a proxy rather than directly by the patient, which may limit the reliability of the results, especially in items regarding covert behaviors [28]. Patients were directed by their HCP, who was a participating physician at one of the study sites, to download the application. This process may introduce a selection bias for patients who already have an HCP, and for patients who show an interest in improving their health by using a digital health application. Lastly, patients need to understand English or Spanish to participate, which may limit the study population.

## 5 Conclusions

The C4T CAD‐PAD optimization study will provide valuable information on the use of the digital health application for patients with CAD and/or PAD to track their medication adherence and other health and lifestyle information related to their risk of cardiovascular disease. The application is widely available in the optimization study, which may help to reduce economic and ethnic disparities in disease management.

## Ethics Statement

Before data collection, the study protocol was approved by the independent ethics committee or institutional review board of each participating site, and patients were fully informed of the observational nature of the study and provided their consent to participate.

## Consent

All patients must sign an informed consent form (ICF) allowing data reporting and collection in accordance with local requirements and/or the study protocol.

## Conflicts of Interest

Dhanunjaya Lakkireddy reports receiving honoraria and/or consulting fees from Abbott, AtriCure, Medtronic, Johnson & Johnson, AltaThera, and Kiniksa Pharma. Dominick J. Angiolillo reports receiving consulting fees or honoraria from Abbott, Amgen, AstraZeneca, Bayer, Biosensors, Boehringer Ingelheim, Bristol Myers Squibb, Chiesi, Daiichi Sankyo, Eli Lilly, Faraday, Haemonetics, Janssen, Merck, Novartis, Novo Nordisk, PhaseBio, PLx Pharma, Pfizer, Sanofi, and Vectura, outside of the submitted work; and declares his institution has received research grants from Amgen, AstraZeneca, Bayer, Biosensors, CeloNova, CSL Behring, Daiichi Sankyo, Eisai, Eli Lilly, Faraday, Gilead, Idorsia, Janssen, Matsutani Chemical Industry Co., Merck, Novartis, and the Scott R. MacKenzie Foundation. Kristofer Charlton‐Ouw reports consulting for W.L. Gore & Associates and VenoStent; and research funding from Inari Medical, Vesper Medical, BD, Johnson & Johnson, and Piomic Medical. Mohannad Bisharat reports consulting for Abbott, Medtronic, BD, Philips, Inari, and Argon. Ante Harxhi, Simrati Kaul, Evelyne Michaud, Stephanie Juan, and CV Damaraju are employees of Janssen Scientific Affairs, LLC. Breeana Woods is an employee of Johnson & Johnson Technology Solutions. Gregory Fontana reports consulting for Abbott, Medtronic Inc., and JenaValve; being a principal investigator for Abbott and a co‐investigator for Edwards; serving on global scientific advisory boards for Abbott, Medtronic Inc., and JenaValve; and serving on a steering committee for Janssen Scientific Affairs, LLC. Marc P. Bonaca reports being the Executive Director of CPC, a nonprofit academic research organization affiliated with the University of Colorado, that receives or has received research grant/consulting funding between August 2021 and present from Abbott Laboratories, Agios Pharmaceuticals Inc., Alexion Pharma, Alnylam Pharmaceuticals Inc., Amgen, Angionetics Inc., Anthos Therapeutics, ARCA Biopharma Inc., Array BioPharma Inc., AstraZeneca and affiliates, Atentiv LLC, Audentes Therapeutics Inc., Bayer and affiliates, Beth Israel Deaconess Medical Center, Better Therapeutics Inc., Boston Clinical Research Institute, Bristol Myers Squibb, Cambrian Biopharma Inc., Cardiol Therapeutics Inc., CellResearch Corp., Cleerly Inc., Cook Regentec LLC, CSL Behring LLC, Eidos Therapeutics Inc., EP Trading Co. Ltd., EPG Communication Holdings Ltd., Epizon Pharma Inc., Esperion Therapeutics Inc., Everly Well Inc., Exicon Consulting Pvt. Ltd., Faraday Pharmaceuticals Inc., Foresee Pharmaceuticals Co. Ltd., Fortress Biotech Inc., HDL Therapeutics Inc., HeartFlow Inc., Hummingbird Bioscience, Insmed Inc., Ionis Pharmaceuticals, IQVIA Inc., Janssen and affiliates, Kowa Research Institute Inc., Kyushu University, Lexicon Pharmaceuticals Inc., Medimmune Ltd., Medpace, Merck and affiliates, Nectero Medical Inc., Novartis, Pharmaceuticals Corp., Novo Nordisk Inc., Osiris Therapeutics Inc., Pfizer Inc., PhaseBio Pharmaceuticals Inc., PPD Development, LP, Prairie Education and Research Cooperative, Prothena Biosciences Limited, Regeneron Pharmaceuticals Inc., Regio Biosciences Inc., Saint Luke's Hospital of Kansas City, Sanifit Therapeutics S.A., Sanofi‐Aventis Groupe, Silence Therapeutics PLC, Smith & Nephew plc, Stanford Center for Clinical Research, Stealth BioTherapeutics Inc., State of Colorado Cancer, Cardiovascular and Pulmonary Disease (CCPD) Grant, The Brigham & Women's Hospital Inc., The Feinstein Institutes for Medical Research, Thrombosis Research Institute, University of Colorado, University of Pittsburgh, VarmX, Virta Health Corporation, Worldwide Clinical Trials Inc., WraSer, LLC, and Yale Cardiovascular Research Group. The other authors declare no conflicts of interest.

## Data Availability

No data are reported in this article.

## References

[1] R. Bauersachs , U. Zeymer , J. B. Brière , C. Marre , K. Bowrin , and M. Huelsebeck , “Burden of Coronary Artery Disease and Peripheral Artery Disease: A Literature Review,” Cardiovascular Therapeutics 2019 (2019): 8295054. 32099582 10.1155/2019/8295054 PMC7024142

[2] S. S. Martin , A. W. Aday , Z. I. Almarzooq , et al., “2024 Heart Disease and Stroke Statistics: A Report of US and Global Data From the American Heart Association,” Circulation 149 (2024): e347–e913. 38264914 10.1161/CIR.0000000000001209 PMC12146881

[3] P. Poredoš and B. Jug , “The Prevalence of Peripheral Arterial Disease in High Risk Subjects and Coronary or Cerebrovascular Patients,” Angiology 58 (2007): 309–315. 17626985 10.1177/0003319707302494

[4] J. A. Gutierrez , H. Mulder , W. S. Jones , et al., “Polyvascular Disease and Risk of Major Adverse Cardiovascular Events in Peripheral Artery Disease: A Secondary Analysis of the EUCLID Trial,” JAMA Network Open 1 (2018): e185239. 30646395 10.1001/jamanetworkopen.2018.5239 PMC6324381

[5] H. L. Gornik , H. D. Aronow , P. P. Goodney , et al., “2024 ACC/AHA/AACVPR/APMA/ABC/SCAI/SVM/SVN/SVS/SIR/VESS Guideline for the Management of Lower Extremity Peripheral Artery Disease: A Report of the American College of Cardiology/American Heart Association Joint Committee on Clinical Practice Guidelines,” Circulation 149 (2024): e1313–e1410. 38743805 10.1161/CIR.0000000000001251 PMC12782132

[6] S. S. Virani , L. K. Newby , S. V. Arnold , et al., “2023 AHA/ACC/ACCP/ASPC/NLA/PCNA Guideline for the Management of Patients With Chronic Coronary Disease: A Report of the American Heart Association/American College of Cardiology Joint Committee on Clinical Practice Guidelines,” Circulation 148 (2023): e9–e119. 37471501 10.1161/CIR.0000000000001168

[7] F. Demsas , M. M. Joiner , K. Telma , A. M. Flores , S. Teklu , and E. G. Ross , “Disparities in Peripheral Artery Disease Care: A Review and Call for Action,” Seminars in Vascular Surgery 35 (2022): 141–154. 35672104 10.1053/j.semvascsurg.2022.05.003 PMC9254894

[8] A. W. Aday and K. Matsushita , “Epidemiology of Peripheral Artery Disease and Polyvascular Disease,” Circulation Research 128 (2021): 1818–1832. 34110907 10.1161/CIRCRESAHA.121.318535 PMC8202714

[9] K. Matsushita , Y. Sang , H. Ning , et al., “Lifetime Risk of Lower‐Extremity Peripheral Artery Disease Defined by Ankle‐Brachial Index in the United States,” Journal of the American Heart Association 8 (2019): e012177. 31500474 10.1161/JAHA.119.012177 PMC6818002

[10] C. A. Kalbaugh , B. Witrick , L. B. Sivaraj , et al., “Non‐Hispanic Black and Hispanic Patients Have Worse Outcomes Than White Patients Within Similar Stages of Peripheral Artery Disease,” Journal of the American Heart Association 11 (2022): e023396. 34927446 10.1161/JAHA.121.023396 PMC9075215

[11] P. A. Soden , S. L. Zettervall , S. E. Deery , et al., “Black Patients Present With More Severe Vascular Disease and a Greater Burden of Risk Factors Than White Patients at Time of Major Vascular Intervention,” Journal of Vascular Surgery 67 (2018): 549–556.e3. 28951156 10.1016/j.jvs.2017.06.089 PMC5794625

[12] S. Arya , Z. Binney , A. Khakharia , et al., “Race and Socioeconomic Status Independently Affect Risk of Major Amputation in Peripheral Artery Disease,” Journal of the American Heart Association 7 (2018): e007425. 29330260 10.1161/JAHA.117.007425 PMC5850162

[13] K. Churchwell , M. S. V. Elkind , R. M. Benjamin , et al., “Call to Action: Structural Racism as a Fundamental Driver of Health Disparities: A Presidential Advisory From the American Heart Association,” Circulation 142 (2020): e454–e468. 33170755 10.1161/CIR.0000000000000936

[14] “PAD National Action Plan,” American Heart Association, accessed April 30, 2024, https://professional.heart.org/-/media/PHD-Files-2/Science-News/p/PAD-National-Action-Plan.

[15] E. M. Spaulding , F. A. Marvel , M. A. Lee , et al., “Corrie Health Digital Platform for Self‐Management in Secondary Prevention After Acute Myocardial Infarction,” Circulation: Cardiovascular Quality and Outcomes 12 (2019): e005509. 31043065 10.1161/CIRCOUTCOMES.119.005509 PMC6697167

[16] A. Abernethy , L. Adams , M. Barrett , et al., “The Promise of Digital Health: Then, Now, and the Future,” NAM Perspectives. Discussion Paper (Washington, DC: National Academy of Medicine). (2022). 10.31478/202206e. PMC9499383 36177208

[17] K. J. Thomas Craig , N. Fusco , K. Lindsley , et al., “Rapid Review: Identification of Digital Health Interventions in Atherosclerotic‐Related Cardiovascular Disease Populations to Address Racial, Ethnic, and Socioeconomic Health Disparities,” Cardiovascular Digital Health Journal 1 (2020): 139–148. 35265886 10.1016/j.cvdhj.2020.11.001 PMC8890337

[18] R. N. Devani , A. Kirubakaran , and M. Molokhia , “Digital Health RCT Interventions for Cardiovascular Disease Risk Reduction: A Systematic Review and Meta‐Analysis,” Health and Technology 12 (2022): 687–700. 35350665 10.1007/s12553-022-00651-0 PMC8947848

[19] E. C. Lee , A. L. Whitehead , R. M. Jacques , and S. A. Julious , “The Statistical Interpretation of Pilot Trials: Should Significance Thresholds be Reconsidered?” BMC Medical Research Methodology 14 (2014): 41. 24650044 10.1186/1471-2288-14-41 PMC3994566

[20] G. A. Lancaster , S. Dodd , and P. R. Williamson , “Design and Analysis of Pilot Studies: Recommendations for Good Practice,” Journal of Evaluation in Clinical Practice 10 (2004): 307–312. 15189396 10.1111/j..2002.384.doc.x

[21] J. Hsia , N. L. Guthrie , P. Lupinacci , et al., “Randomized, Controlled Trial of a Digital Behavioral Therapeutic Application to Improve Glycemic Control in Adults With Type 2 Diabetes,” Diabetes Care 45 (2022): 2976–2981. 36181554 10.2337/dc22-1099 PMC9862458

[22] J. N. Rasmussen , A. Chong , and D. A. Alter , “Relationship Between Adherence to Evidence‐Based Pharmacotherapy and Long‐Term Mortality After Acute Myocardial Infarction,” JAMA 297 (2007): 177–186. 17213401 10.1001/jama.297.2.177

[23] P. M. Ho , C. L. Bryson , and J. S. Rumsfeld , “Medication Adherence: Its Importance in Cardiovascular Outcomes,” Circulation 119 (2009): 3028–3035. 19528344 10.1161/CIRCULATIONAHA.108.768986

[24] P. M. Ho , D. J. Magid , S. M. Shetterly , et al., “Medication Nonadherence Is Associated With a Broad Range of Adverse Outcomes in Patients With Coronary Artery Disease,” American Heart Journal 155 (2008): 772–779. 18371492 10.1016/j.ahj.2007.12.011

[25] L. M. Shah , J. Ding , E. M. Spaulding , et al., “Sociodemographic Characteristics Predicting Digital Health Intervention Use After Acute Myocardial Infarction,” Journal of Cardiovascular Translational Research 14 (2021): 951–961. 33999374 10.1007/s12265-021-10098-9 PMC8127845

[26] “Mobile Fact Sheet,” Pew Research Center, accessed August 21, 2024, https://www.pewresearch.org/internet/fact-sheet/mobile/.

[27] J. Nicosia , A. J. Aschenbrenner , S. L. Adams , et al., “Bridging the Technological Divide: Stigmas and Challenges With Technology in Digital Brain Health Studies of Older Adults,” Frontiers in Digital Health 4 (2022): 880055. 35574256 10.3389/fdgth.2022.880055 PMC9098948

[28] A. Lopez , L. Tinella , A. Caffò , and A. Bosco , “Measuring the Reliability of Proxy Respondents in Behavioural Assessments: An Open Question,” Aging Clinical and Experimental Research 35 (2023): 2173–2190. 37540380 10.1007/s40520-023-02501-z PMC10520105

